# Promoting trustworthy file sharing: A community-governed dApp approach

**DOI:** 10.1371/journal.pone.0337697

**Published:** 2025-12-30

**Authors:** Chong-Gee Koa, Swee-Huay Heng, Ji-Jian Chin

**Affiliations:** 1 Faculty of Information Science and Technology, Multimedia University, Melaka, Malaysia; 2 School of Engineering, Computing and Mathematics (Faculty of Science and Engineering, University of Plymouth), Plymouth, United Kingdom; Beijing Technology and Business University, CHINA

## Abstract

In the cyber digital world, file sharing has become a cornerstone of global connectivity, facilitating the exchange of media, information, and resources among people worldwide. File sharing is already a norm in our daily lives, with many existing platforms allowing users to download shared content without verification or with only basic verification. Hence, the trustworthiness of the shared content is a concern. Meanwhile, blockchain technology has evolved over the last 15 years since its introduction, renowned for its decentralised, secure, and transparent system enabling efficient transactions, traceability across various industries. To address the mentioned open issue, we introduce a decentralised application (dApp) called Filetherst which is backed by ETHERST, a blockchain-based PKI. Filetherst focuses on ensuring shared files are trustworthy through the involvement of the virtual community in evaluating trustworthiness through actions triggered by community members. The foundational principle lies in encouraging users to actively take part in governing the platform, fostering a culture of transparency and accountability, preventing users from using multiple fake accounts to create false trustworthiness. Filetherst is the first dApp that leverages the Ethereum ERC-20 token to establish a self-governing network aimed at addressing the dissemination of harmful and inappropriate content within a virtual community.

## Introduction

In the digital world, symmetric and asymmetric encryption schemes are used for authentication within a public key infrastructure (PKI). With PKI, various online applications can perform different operations in a secure context. Without knowing the person who is operating at the other end in a virtual world, the existence of PKI is a crucial to ensure we are in a secure digital communication. Meanwhile, file-sharing serves as a fundamental pillar of global connectivity that enables the seamless exchange of media, information, and resources across borders. However, as file-sharing has become increasingly ingrained in our daily routines, concerns about the integrity and trustworthiness of shared content have arisen. Many existing platforms offer little to no verification processes, leaving users doubt about the authenticity of the files they access, which may potentially contain copyrighted or inappropriate material.

In this paper, we present Filetherst, a dApp designed to ensure the trustworthiness of shared files through community-driven evaluations that address the above concerns. Built on the foundational principles of transparency and accountability, Filetherst leverages ETHERST [1], a blockchain-based PKI that incorporates incentivisation and disincentivisation mechanisms proven effective through simulations of various node behaviours. By integrating the Interplanetary File System (IPFS) and being backed by ETHERST, Filetherst mitigates the risk of fraudulent activities, such as sharing copyrighted materials through multiple fake accounts. Through the Filetherst platform, users are empowered to participate in the governance of shared content by casting trust or untrust votes, thereby fostering a self-regulating ecosystem in which community-evaluated trustworthiness guides user interactions with shared files. Filetherst inherits the advantages of ETHERST, effectively mitigates Sybil attacks, and encourages the growth of virtual communities distinguishing itself from other existing proposals.

## Preliminary

### Blockchain

Essentially, blockchain is a decentralised and distributed ledger system that records transactions across a network of computers. Unlike traditional centralised databases, blockchain operates on a peer-to-peer network, where each transaction is securely encrypted and linked to the preceding one, forming an immutable chain of blocks.

First conceptualised by Satoshi Nakamoto in 2008 as the underlying technology of Bitcoin, blockchain has since evolved into a versatile platform with applications spanning finance, supply chain management, healthcare, and beyond [2]. Its decentralised nature eliminates the need for intermediaries, facilitating transparent and tamper-proof transactions while ensuring data integrity and security.

By enabling trustless and transparent interactions, blockchain technology promises to revolutionise industries, streamline processes, and empower individuals worldwide. As we delve deeper into its potential, the transformative impact of blockchain on the digital world becomes increasingly apparent. Therefore, blockchain technology is a key component of Web 3.0, the next phase of the Internet. It aligns with Web 3.0’s goals of decentralisation, user empowerment, and enhanced security [3].

In two separate studies on the application of blockchain technology, Lu and Chen et al. observed that blockchain has already been implemented in various domains, including transactions and payments, the Internet of Things (IoT), e-government and education, healthcare, privacy and data management, as well as 5G and 6G networks [4,5]. The potential of blockchain technology continues to draw significant research interest, with numerous studies conducted to explore its applications and implications [6–10].

### Ethereum

Ethereum [11] is a decentralised, open-source blockchain platform that went live in 2015 and enables developers to build and deploy smart contracts and decentralised applications (dApps). Its native cryptocurrency, Ether (ETH), acts as the fuel for network operations, underpinning the execution of smart contracts and dApps. Ethereum’s pioneering feature lies in its ability to host and execute complex smart contracts, setting it apart from traditional cryptocurrencies like Bitcoin. This capability has given rise to a wide array of applications, from decentralised finance platforms to non-fungible token marketplaces, driving innovation in the blockchain space.

Moreover, Ethereum is currently undergoing a major upgrade known as Ethereum 2.0, aimed at addressing the network’s scalability, security, and sustainability. This upgrade involves transitioning from a proof-of-work (PoW) to a proof-of-stake (PoS) consensus algorithm, implementing sharding, and introducing other optimisations to enhance efficiency. The ongoing development efforts in Ethereum 2.0 reflect the platform’s commitment to evolving to meet the increasing demand for decentralised applications and smart contracts. This transition holds the potential to bring about significant improvements, making Ethereum more adaptable and sustainable in the rapidly growing blockchain landscape.

Ethereum has significantly impacted the blockchain and cryptocurrency ecosystem by providing a robust foundation for the creation of innovative decentralised solutions. Its smart contract functionality, coupled with the ongoing evolution towards Ethereum 2.0, positions it as a leading force in driving the advancement of decentralised technologies and applications.

### Infura

Infura is a blockchain infrastructure service that provides scalable and reliable access to the Ethereum network and other Web3 protocols without requiring developers to operate their own nodes. It enables tasks such as deploying smart contracts, querying transactions, and monitoring events without the overhead of maintaining blockchain infrastructure [12].

As a node-as-a-service provider, Infura maintains high-availability node clusters and abstracts the complexity of synchronisation, upgrades, and resource management. This makes it a widely adopted solution, powering applications such as MetaMask [13] and Truffle, and serving as a critical component in the Ethereum ecosystem.

Alternative providers include Alchemy [14] and QuickNode [15], which offer similar services with added features like analytics and performance monitoring.

### InterPlanetary File System (IPFS)

In the evolving landscape of digital communication and information sharing, traditional methods of storing and accessing data face challenges such as centralisation, inefficiency, and vulnerability to censorship. In response to these shortcomings, the IPFS emerges as a groundbreaking solution, offering a decentralised and distributed approach to file storage and retrieval.

IPFS, introduced by Juan Benet in 2014 [16], operates on a peer-to-peer network protocol, enabling users to store and retrieve verifiable data across a multitude of nodes worldwide. Unlike the conventional web’s reliance on centralised servers, IPFS employs a content-addressed system, where files are identified by unique cryptographic hashes. This methodology ensures data integrity, as any alteration to a file would result in a distinct hash, thereby facilitating secure and tamper-evident content distribution.

By harnessing IPFS, individuals and organisations can transcend the limitations of centralised infrastructure, promoting a stronger, faster, and censorship-proof digital environment. With its promise of decentralisation and data sovereignty, IPFS stands as a pioneering force in shaping the future of Internet infrastructure and information exchange.

### Pinata - IPFS API service

Pinata API serves as a gateway to IPFS, providing developers with seamless integration and management capabilities for their decentralised storage needs. With Pinata API, developers can effortlessly upload, pin, and retrieve content on the IPFS network, ensuring data persistence and availability across distributed nodes worldwide [17].

From content creators seeking immutable storage solutions to developers building dApps, Pinata API empowers users to harness the benefits of IPFS with ease and efficiency. Whether it is storing multimedia files, website assets, or application data, Pinata API offers a reliable and scalable solution for leveraging the decentralised power of IPFS in diverse use cases.

### ETHERST - The information trustworthiness blockchain-based PKI

Before we go in deeper to our proposed dApp solution, we visit the backend trustworthiness framework that we used - ETHERST. ETHERST introduced a novel approach to blockchain-based PKI, incorporating a system of rewards and penalties atop the conventional distributed PKI [1]. Operating on the Ethereum blockchain, which was conceptualised by Buterin [11], ETHERST leveraged Ethereum’s unique functionality, particularly its support for executing code through “smart contracts”. These smart contracts empower developers to create dynamic logic that harnesses the capabilities of blockchain technology.

ETHERST comprises two smart contracts:

1. PKIToken.
2. ETHERST.

With the logic in PKIToken smart contract, a new token that conforms to the ERC-20 standard [18], with the same name “PKIToken” was created and served as the incentive mechanism within the ETHERST ecosystem, rewarding or penalising network nodes based on their behaviours. The core logic of ETHERST was embedded within the ETHERST smart contract. Through ETHERST, users can generate data on the blockchain and authenticate it using their private keys, asserting ownership within the community. Diverging from conventional distributed PKI models like the Web of Trust (WoT) [19], ETHERST introduced features enabling users to establish or withdraw trust from signed data. Upon joining ETHERST, users receive an initial allocation of PKIToken, which they can utilise to validate or invalidate other users’ signed data. The distribution and redistribution of PKIToken among users are determined by algorithms tracking their trust-related actions within the network. With the features that come with ETHERST, we utilised it as an information trustworthiness backend framework for Filetherst that we will present in the following sections.

PKIToken is an ERC-20 token [18] implemented by ETHERST to reward and punish nodes based on their good and bad actions within the ETHERST network. The core logic of the blockchain-based PKI for ETHERST is found in its smart contract. ETHERST allows users to create data on the blockchain and sign it with their private key, asserting that the information was originally created and published by them within the community. Additionally, users have the ability to revoke the signatures they have applied to specific data. Beyond the conventional distributed public key infrastructure like the WoT [19], ETHERST offers functionalities that enable users to “trust” or “untrust” signed data. Upon joining ETHERST, users are pre-allocated PKITokens, which they can use to establish trust in or untrust the signed data of others. An algorithm governs the distribution of PKITokens, adjusting the amount a user owns based on their “trust” and “untrust” actions. Furthermore, the ETHERST smart contract contains logic to prevent Sybil attacks by imposing delays on “trust” and “untrust” actions for each node [1]. Each user has a unique delay period *T* established by ETHERST. If a user attempts to perform a “trust” or “untrust” action within their designated delay period *T*, that action will fail. The value of *T* is increased if a user’s subsequent action occurs before the end of the delay period, preventing dishonest behavior such as repeatedly trusting self-registered dishonest accounts or unjustly untrusting others. With these features, we utilise ETHERST as an information trustworthiness backend framework for Filetherst, which we will present in the following sections.

## Trustworthiness issues of file-sharing

Sharing files over the Internet is a means to exchange information in this era. File-sharing over the Internet can be done via different methods nowadays — for instance, file-sharing with messaging applications such as WhatsApp and Telegram. Besides, sharing files with cloud storage services like Google Drive and DropBox is another method that is commonly used by Internet users. Another popular method of file-sharing is peer-to-peer (P2P). Unlike the first and second methods, P2P file-sharing is a decentralised method which does not have a centralised party that governs the sharing actions. The popular P2P networks are BitTorrent [20] and eDonkey [21]. With the invention of IPFS [22] in 2014, file-sharing entered into a new P2P technology era.

With the popularity of file-sharing via Internet, people are starting to look into the problem of copyright infringement and malicious files. Anybody can share a copyrighted file to the network and share it with another person from all over the world. In fact, the file-sharing copyright infringement problem has been studied by researchers for many years either from the aspect of law or information technology, for instance, Fung and Lakhani discussed the impact of P2P file-sharing and copyright infringement. Fung and Lakhani examined copyright violations in P2P file-sharing and current anti-piracy efforts. They explored international developments, trends, issues, and solutions related to P2P file-sharing of copyrighted material. The analysis includes an overview of P2P file-sharing, its significance in the digital media economy, and legal challenges. Comparative analysis covers global efforts to prevent digital piracy, including legislation, case law, and practices in various jurisdictions such as the US, UK, France, Sweden, Japan, and Hong Kong. The article identifies deficiencies in Hong Kong’s legal framework for addressing P2P copyright infringement. Their research concluded with insights into existing preventive measures and suggested future legal and non-legal strategies for combating digital piracy globally and in Hong Kong [23].

In this paper, we are looking into how the currency property of blockchain technology can be utilised to build a self-govern network to cooperate to combat malicious and improper content sharing over the IPFS. In this research, our contribution is the introduction of the first decentralised file-sharing platform that incorporates the shared-content trustworthiness evaluation and self-governance by the community members. Before we continue in detail with our proposal, we would like to go through some efforts from researchers on file-sharing with the blockchain technology in the next section and the research gap.

## Related work

The emergence of decentralised P2P file-sharing systems introduced novel challenges, particularly concerning the stability and efficiency of the network. With the potential threat posed by egoistic nodes capable of destabilising the system, researchers turned to incentive mechanisms rooted in blockchain technology to incentivise cooperation among network participants. Vimal and Srivatsa looked into the proposed method outlined in recent research, aiming to enhance the efficiency of P2P file-sharing systems through the integration of IPFS and blockchain [24]. The approach emphasised aspects of trustworthiness and proximity awareness during file transfers, addressing critical concerns in autonomous distributed environments. Furthermore, the review discussed the inherent issues faced by blockchain technology and explored the potential solutions offered by IPFS. IPFS, characterised by its cluster of hashed files distributed across network nodes, facilitated efficient resource sharing by allowing users to retrieve files through simple hash calls. To address the high-throughput challenge encountered by individual IPFS users, the concept of Filecoin was introduced, wherein miners were incentivised to provide storage services on a distributed network of local providers. They also highlighted the discussion on the service provided during file transfer, emphasising security strengths and various incentives associated with IPFS. In this research, they provided a comprehensive overview of the advancements in P2P file-sharing systems, focusing on the convergence of IPFS and blockchain technology to address critical challenges and enhance system efficiency.

Zhao and O’Mahony discussed the challenges artists face regarding copyright infringement and the lack of transparency in royalty distribution in the music industry. As a result of their research, a prototype implementation of a blockchain-based solution was proposed called BMCProtector [25]. The proposed platform allows musicians to upload music and automatically receive royalty payments. It incorporated a digital watermarking system to identify ownership and track unauthorised distributions. Versioning system proposed in BMCProtector eases the copyright owner to update their smart contract as needed, ensuring flexibility in managing copyright terms.

Mehta et al. introduced a blockchain-based platform to allow image sharing and copyright protection with perceptual hashing [26]. The proposed system leverages blockchain technology to create a transparent, secure, and censorship-resistant marketplace for digital images. Unlike traditional marketplaces, this system ensures that each image is uploaded only once, shielding original works from unauthorised copies. The proposal used four most famous perceptual hashing algorithms which are included in the python ImageHash library and they are wavelet hash, difference hash, phash and average hash. The perceptual hashing is used to identify the images uploaded is the first and original version to avoid copy with minimum modification and re-upload as a new image by non original owner. One of the future work suggested was rewards management and remuneration for copyright breach.

Agyekum et al. implemented a new platform for the digital content producers with a decentralised approach to digital media copyright protection, integrating the IPFS and blockchain technology [27]. The platform included a fingerprint generator for image, video and audio with different type of algorithms. The fingerprint is the unique identity for a digital content regardless is an image, a video or an audio. Perceptual hash was used to generate as a fingerprint for an image, and for video, segmentation key-frame dhash algorithm was implemented and finally the fingerprint for audio was generated by dividing audio into several blocks and each blocks underwent a Fourier transformation and then the subscript of the highest energy point was extracted by the molecular band.

Traditional data-sharing platforms typically relied on trusted third parties (TTP), leading to issues such as lack of trust, transparency, security, and immutability. In response to these challenges, Naz et al. reviewed and explored the proposal of a blockchain-based secure data-sharing platform that leveraged the advantages of the IPFS [28]. The proposed platform involved uploading metadata to an IPFS server by the owner, which was then divided into *N* secret shares. Security and access control were enforced through the execution of access roles defined in smart contracts by the owner. User authentication occurred through RSA signatures, followed by the submission of a requested amount as payment for digital content. Upon successful data delivery, users were encouraged to register reviews about the data, which were subsequently validated using Watson analyser to filter out fake reviews. Users providing valid reviews received incentives, thereby maximizing the number of reviews submitted for each file. This approach integrated decentralised storage, Ethereum blockchain, encryption, and incentive mechanisms.

Jaiman et al. explored how to incentivise data sharing in decentralised environments using blockchain technology [29]. They categorised incentivise mechanism into five categories below:

1. Control
2. Research
3. Monetary
4. Reputation
5. Knowledge

In this research, Jaiman et al. examined various compensation models to motivate data sharing, focusing on the monetary incentivisation. An incentivisation model based on LUCE [30] was proposed where there are two parties involved: data provider and data requester. The research introduced a dynamic incentivisation amount and demonstrated that a cost compensation approach can effectively cover data provider expenses while balancing overall incentive, by carrying out simulations and cost analysis.

In the current era, anyone can contribute to sharing information online. While high-quality knowledge files drive societal progress, pirated or low-quality ones hinder it. Presently, organisations typically rely on centralised servers to manage these files, resulting in various issues like opaque transaction processes, copyright infringement, and inconsistent file management standards.

Kang et al. sought to address these challenges by ensuring the secure storage of knowledge files while facilitating efficient and lawful file-sharing [31]. They proposed a novel approach that combines Named Data Network (NDN) technology with distributed blockchain and IPFS. This method utilises NDN for content signature and encryption, effectively separating file security from transmission processes. Additionally, it employed flexible NDN routing strategies and integrated IPFS private storage networks to enhance data storage security.

Furthermore, the method leveraged blockchain consensus mechanisms to facilitate traceable file-sharing among participating nodes. The paper elucidated the method’s structure and principles, detailing the file upload and transfer processes. Through comparative analysis, it evaluated the performance, advantages, and limitations of the proposed method, while also identifying potential avenues for future research and development.

The rapid growth of internet data, driven by cloud-based services, poses challenges for centralised storage providers in ensuring data security, access control, and performance. To address these issues, Han et al. presented an efficient peer-to-peer data storage and sharing system [32]. Leveraging a modified EOSIO blockchain and the IPFS, their solution named FIBPRO, ensured data secrecy through hybrid encryption and enabled multiple uses and persistent storage. Additionally, the expanded blockchain component offers a flexible transaction audit solution, reducing costs and tracing malicious behaviour. Through system analysis and experimental evaluation, FIBPRO achieved a significant reduction in on-chain storage consumption and demonstrates high performance, with a throughput of approximately 1300 TPS, an upload efficiency of 2.31MB/s, and a download rate of 5.29MB/s, showcasing its availability and scalability.

Verma and Kanrar carried out a study to address concerns about data privacy in public cloud storage by proposing a solution that combines blockchain technology and keyword-searchable attribute-based encryption (ABE) [33]. Traditional cryptographic methods assume trust in the cloud service provider, but the need for secure data sharing has prompted new approaches. Blockchain records transactions while the IPFS ensures data privacy and immutability. This model offers low computational costs, attribute revocation, and smart audit contracts. Experimentation on the Ethereum network validated its effectiveness, showing resistance to keyword guessing and “chosen plaintext” attacks.

Son et al. pointed out the growing popularity of decentralised storage services due to their cost-effectiveness, resilience, and privacy benefits, however, the services also attracted malicious actors who engaged in illegal activities such as phishing, malware distribution, and copyright infringement. Traditional investigation methods for cloud and peer-to-peer file-sharing services are inadequate for decentralised storage due to its censorship resistance and decentralisation. To tackle these challenges, a new forensic investigation framework, IF-DSS was proposed, covering identification, collection, examination, analysis, and prevention of further content distribution across nodes, peers, gateways, and Internet areas. They presented case studies involving phishing and large-scale file-sharing using IPFS, and demonstrated the effectiveness and applicability of the IF-DSS framework.

In summary, we noticed that among the above blockchain-based proposals for improving file sharing, most of the research focused on using hashing techniques to manage the genuineness of the content. There has been no direct attempt to tackle the issue of illegal and non-copyrighted material sharing on the Internet, as mentioned in Section Trustworthiness issues of file-sharing. In addition, we found that no proposal leverages the currency property of blockchain to address this open issue, except for Mehta et al., who suggested a reward system in their future work. With the combination of the immutability and trustworthiness of blockchain technology as a foundation, we believe that the currency property of blockchain can be utilised to implement a reward-and-punishment mechanism in a file-sharing platform. This represents a new approach to mitigating the issues of illegal and non-copyrighted material sharing, as well as the spread of malicious files. The Internet community is built by its users, and users are the key to governing illegal activities on the Internet. Therefore, we aim to build a file-sharing platform integrated with a blockchain-based WoT reward-and-punishment framework, named ETHERST [1], to address this gap.

## Methodology

The propose solution is the implementation of a novel reward and punishment system, which aims to incentivise lawful file-sharing practices while discouraging copyright infringement on the platform. The development process includes initial system architecture design, smart contract development for governing the reward and punishment mechanisms, integration of user interfaces for seamless interaction. The file-sharing platform was developed using ETHERST as the underlying technology, to enforce the reward and punishment system, ensuring that all transactions related to file-sharing are securely logged and verified on the blockchain. Simultaneously, the user interface will be designed and implemented to provide a user-friendly experience, enabling seamless interaction with the functionalities of the dApp.

Subsequently, a testing phase was conducted, including the implementation of the proposal on Sepolia testnet and cloud for the dApp to validate the system as a whole. The testing phase involves simulated real-world scenarios to assess the basic functionalities of the implemented reward and punishment mechanisms. Throughout this process, the aim is to provide a comprehensive methodology not only for the development of the dApp but also for the integration of blockchain technology, specifically ETHERST, to address the open issue of file-sharing with copyrighted material and malicious files.

### Dapp development and deployment

In this section, we will outline the development stack and deployment procedures for Filetherst. Beginning the journey of decentralised application development involves installing a development framework as the initial step. TruffleSuite, a popular Ethereum-based development suite [34], fulfils this role by serving as both a testing framework and a conduit to the blockchain via the Ethereum Virtual Machine (EVM). Simplifying developers’ tasks, TruffleSuite provides a comprehensive toolkit for compiling smart contracts into machine language, deploying them onto public or private Ethereum networks, establishing connections with other contracts, and managing their binary code. By employing TruffleSuite’s automated contract testing capabilities, developers can swiftly construct applications while locally simulating the Ethereum network on their machines, facilitating transaction execution without incurring actual costs.

The next step in decentralised application development is coding the smart contract using languages like Solidity. Solidity, an object-oriented, high-level language, is tailored for writing smart contract source code executable on the EVM, boasting syntax akin to JavaScript. Offering object-oriented perks such as inheritance and libraries for reusable code creation, Solidity allows for flexible smart contract development. While various tools can be employed for writing smart contracts, Visual Studio Code (VS Code) stands out as a popular choice due to its integration capabilities with Solidity extensions. Software developers use Solidity to develop the smart contract code, which is then compiled into “EVM bytecode” for interpretation by the Ethereum Virtual Machine. Furthermore, web3.py or any web3 library is essential for code development, serving as a bridge between the frontend application and the Ethereum blockchain. Before smart contacts are deployed, the smart contract’s Application Binary Interface (ABI), which includes all its functions and features, and also metadata of the contract in JSON format, can be generated by the Solidity compiler after the building and compilation of the smart contract code.

The third stage in constructing the decentralised application involves creating the frontend application, which interfaces with the program developed in the previous step. Frontend applications can take various forms, such as web, mobile, or desktop applications. For instance, the decentralised application described in this paper, Filetherst, is a web application developed using the Python programming language and the Django framework [35]. Django is a widely-used open-source web framework renowned for its rapid development capabilities, robust security features, extensive functionality, scalability, and versatility.

The fourth stage of decentralised application development involves deploying the smart contract to a test network and conducting comprehensive testing. Ganache, known for its user-friendly interface, serves as the designated test network for development purposes. Bundled with TruffleSuite, Ganache operates as a personal blockchain, running locally on a developer’s computer and tailored specifically for Ethereum development. With its automated network configuration and provision of ten accounts, each holding 100 ETH along with their corresponding mnemonics, Ganache facilitates the simulation of Ethereum’s expansive network. This functionality allows developers to rigorously test smart contract operations, analyse account balances, and evaluate transaction costs. Furthermore, developers can experiment with the balances of the ten accounts to simulate various scenarios, ensuring the concrete functionality of the smart contract. Consequently, developers can test smart contracts efficiently, without incurring any costs and with significantly faster transaction execution speeds compared to the mainnet and public testnets like Goerli or Sepolia.

The final stage in decentralised application development entails deploying the entire application onto the Ethereum mainnet, making it accessible to the public. However, prior to deployment, the application must undergo compilation, and the developer should possess Ethereum’s currency, Ether, in their account. This is because deploying a smart contract involves an Ethereum transaction, with each transaction incurring a gas fee payable in Ether. Infura [12], serving as a gateway and web3 provider, can be utilised to consume Ethers and transfer the local executable to the main Ethereum network. In the post-deployment stage, the main Ethereum network will return the contract account address and the application’s frontend component requires an update. The frontend component itself needs to be deployed on a server for full decentralisation.

**Fig 1:**
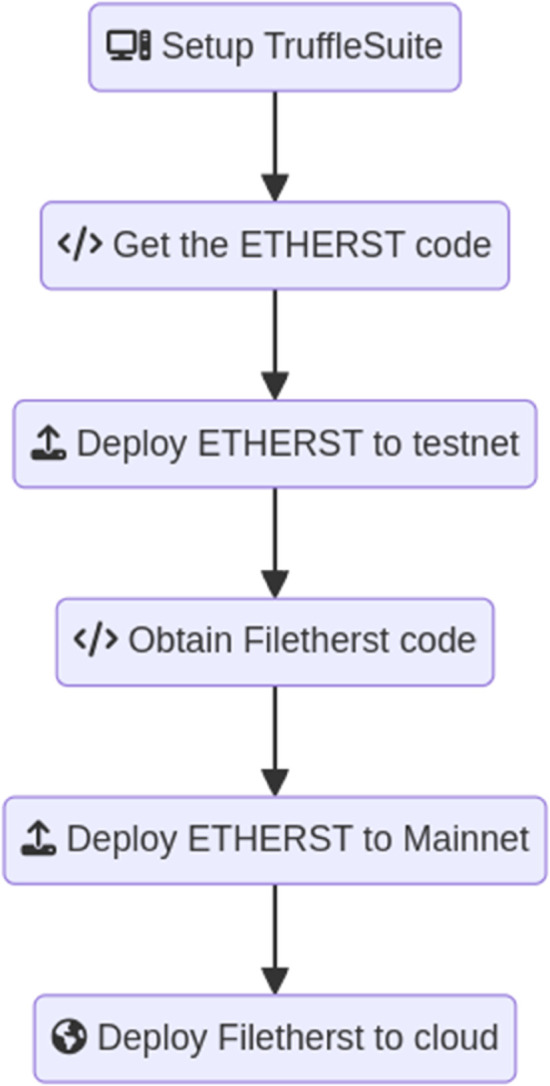
Steps to implement Filetherst.

Fig 1, as depicted below, summarises the steps to develop and deploy an Ethereum decentralised application, using Filetherst as an example, and shows some of the tools used for the development and deployment. Filetherst was developed following the aforementioned methodology and the main Ethereum dApp layers below are the foundation of it.

- Smart contract: This is the layer defining the logic and rules of the dApp, and the code of smart contract is written in Solidity programming language which will be deployed on the Ethereum network afterwards. Subsequently, the deployed smart contracts will be executed by the Ethereum Virtual Machine (EVM).
- Application: This layer consists of 2 main items, the business logic that interacts with the smart contract and the user interface (UI). The communication with Ethereum network is via an API, and the UI maybe in the form of either a mobile application or a web application.
- Ethereum network: Layer that comprises nodes that join and operate in Ethereum and help to validate transactions, forming a distributed network responsible for maintaining the integrity of blockchain.

After deployment, blockchain explorers like Etherscan can be used for application’s performance monitoring. This analytical platform provides comprehensive insights into public Ethereum data, enabling real-time tracking of transactions, blocks, wallet addresses, smart contracts, and a variety of other metrics.

## Using Filetherst

We recommend the integration of ETHERST in the distribution of verification processes by community members of the file-sharing platform. The idea of integration is as below:

1. Create a file-sharing platform with Python programming language and Django Web framework.
2. File-sharing actions via IPFS with Pinata API.
3. Keep file information in ETHERST as attributes.
4. File sharer signed on the attributes.
5. Community members to trust or untrust on the attributes.
6. The result of community trust or untrust actions from ETHERST determines the trustworthiness of the file shared (whether it is a legal material).

**Fig 2:**
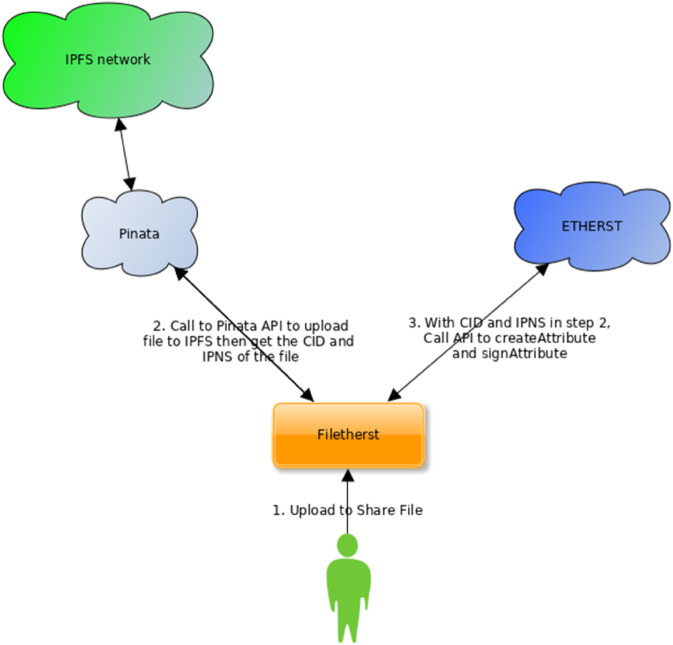
Architecture of Filetherst.

We have developed a prototype dApp with the above idea, and it is named Filetherst. Fig 2 depicts the details of technical steps involved in sharing a file through uploading to IPFS and creating a corresponding record in ETHERST.

**Fig 3:**
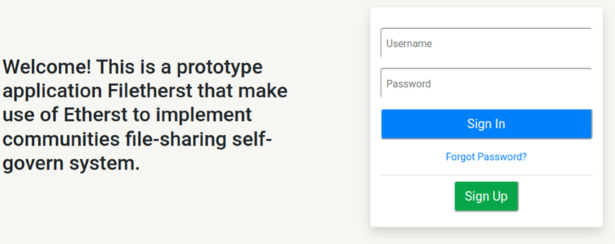
Home page of Filetherst.

**Fig 4:**
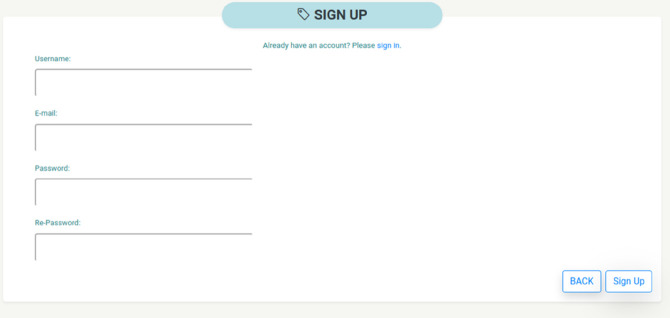
Signup page of Filetherst.

In the following paragraphs, we are going to discuss the proposed idea using Filetherst to have a better understanding. We begin with the home page as shown in Fig 3. As the first step, we begin with signing up a user account. Fig 4 shows the sign up page for Filetherst. Users are required to input the username, email and secret password together with a reconfirmed password to sign up.

Whenever a user signs up successfully, Filetherst will trigger ETHERST to create an account on the Ethereum blockchain which is also known as the wallet. Upon successful account creation, ETHERST will allocate 50 PKIToken as an initial amount. The PKIToken is needed in the process of trusting and untrusting the file shared by others. To demonstrate the entire process, we start with the registration of a user with the username “testuser”.

**Fig 5:**
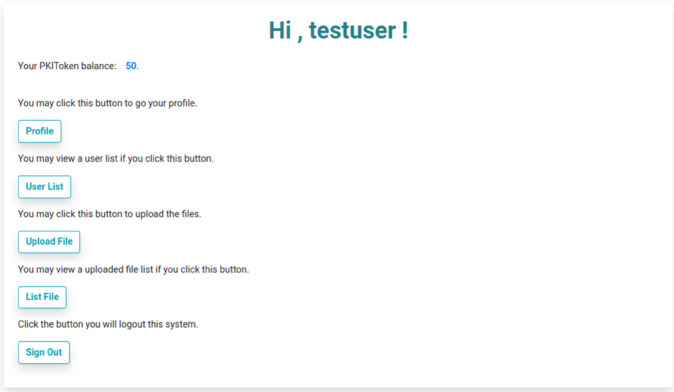
User’s dashboard page in Filetherst.

Upon signing up, “testuser” will be forwarded to the dashboard page and the user is linked to other pages via buttons. In addition, PKIToken balance of the user will be shown in this dashboard page as presented in Fig 5. As mentioned above, all newly registered users will be provided 50 PKIToken as the initial amount allocated by the ETHERST framework.

From the dashboard page, user “testuser” can enter the upload file page to start sharing files in the community, he can upload a file and the Filetherst will automatically upload the file to IPFS via Pinata API. At the same time, it will create an attribute on ETHERST and “sign” on it to certify the file shared is genuine. However, whether the file is genuine and non-copyrighted is still unknown. When the file is uploaded, the flow behind occurs, the frontend will make a call to Pinata API to upload the file to IPFS and the API will respond with status and an IPFS uri that linked to the file. Subsequently, Filetherst will call the function “createAttribute” in ETHERST smart contract and create corresponding data inside blockchain. Finally, Filetherst will call the function “signAttribute” function in ETHERST smart contract to sign on the attribute just created, as a step to declare the genuineness of the file to the community.

**Fig 6:**
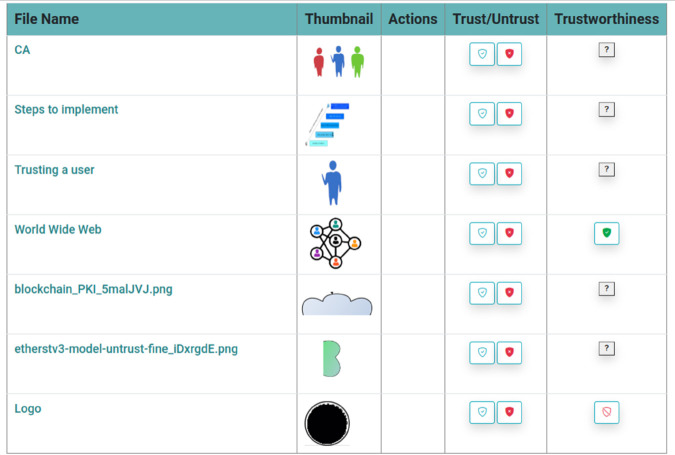
File listing page.

After a file is uploaded and “signed”, it is opened to the community to “trust” or “untrust” this file. As shown in Fig 6, the user “hello” browses to the file list page, and all the files uploaded by the community are listed. User “hello” can “trust” the files uploaded by others if he confirms the file is genuine. In contrast, if he believes that the file infringes the copyright of others, an “untrust” action can be applied to the file.

If user “hello” opts to “trust” on file “World Wide Web” in the file listing page as depicted in Fig 6, Filetherst will trigger the function “trustSignature” on EHTHERST smart contract. Oppositely, if the user “hello” chooses to “untrust” on file “Word Wide Web”, Filetherst will trigger the function “untrustSignature” on ETHERST smart contract. ETHERST will collect the trust and untrust actions from the community and compute the trustworthiness of the file. ETHERST will operate according to its internal algorithm to evaluate the trustworthiness based on other users’ actions.

**Fig 7:**
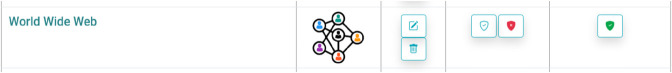
A file has been trusted.

**Fig 8:**
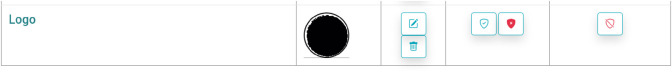
A file has been untrusted.

Column “Trustworthiness” is the column that displays the result through the ETHERST based on the internal algorithm whether a file is “trusted” or “untrusted” by the community members. An ETHERST-trusted file will be displayed as a “green shield” icon under the column “Trustworthiness” on the file listing page, as shown in Fig 7. On the other hand, an ETHERST-untrusted file will be represented by a “red-slashed-shield” icon as depicted in Fig 8. An icon of a “question mark” means the ETHERST still does not have enough “trust” or “untrust” actions to compute the trustworthiness of this file.

The community members can observe the value of “Trustworthiness” to evaluate whether a shared file is trustworthy. When sharing copyrighted content in Filetherst, the user who shared them will be penalised. The PKI token balances will be deducted based on ETHERST internal algorithm and if the PKIToken balance is below 0, the user will be blocked from using Filetherst. The information on trustworthiness will help the community keep the originality and genuineness of the file shared.

## Discussion

### System requirements, limitation and implementation of Filetherst

We have implemented Filetherst on a development machine in our research, the detail of the hardware and software are shown as below

1. Machine: MSI GF66-12UGS - 12th Gen Intel^®^ Core™ i7-12700H and 64G RAM
2. Operating System: Ubuntu Jammy Jellyfish (22.04)
3. Development tools: TruffleSuite, Django framework
4. Blockchain: Ganache blockchain
5. Smart Contracts: ETHERST smart contracts
6. Programming Language: Solidity, Javascript, Python

Simulations of random activities have been carried out with different total numbers of members, and we found that with the above system specifications, the maximum number of concurrent members is about 50, with the bottleneck occurring at the Ganache blockchain, which is reasonable. In addition, we also examined the scalability of Filetherst, since scalability is one of the keys to success in a production system. The scalability of Filetherst depends on the scalability of each component that comprises it. Firstly, the Filetherst frontend is developed with the Django framework, which is well known for its scalability [36]. Subsequently, the data is uploaded to IPFS, which is scalable through the use of IPFS Cluster [37]. Finally, the Filetherst backend — ETHERST, is implemented on the Ethereum blockchain, where scalability is guaranteed.

While the simulations were carried out on a local development machine with Ganache, we also deployed the Filetherst backend — ETHERST on the Sepolia testnet, after collecting sufficient Ether via the Sepolia faucet, to evaluate the scalability of the Filetherst platform in practice. The ETHERST smart contract for Filetherst was deployed on the Sepolia network and is viewable at https://sepolia.etherscan.io/address/0xbf64c4cad8b5c86e89be996431d73d3c90d3fede. On the other hand, Filetherst frontend was deployed on cloud and viewable at https://etherstweb.v-aim.com.

The deployment costs are tabulated in Table 1.

**Table 1:** Costs of Filetherst deployment.

Component	Cost (ETH)	Cost (USD)
ETHERST version 3.0	0.011607392532500699 [1]	52.04 ^1^
Filetherst frontend	-	8.47 ^2^

^1^ One time fee, based on ETH to USD conversion rate in Aug 2025.

^2^ Estimated hosting fee on Amazon Web Service (AWS) EC2 t2.micro per month.

The Filetherst platform is composed of two components, and the total cost of deployment is determined by aggregating the deployment costs of each individual component. The deployment cost for ETHERST version 3.0 amounts to 0.011607392532500699 ETH [1]. The Filetherst frontend constitutes a web application that requires deployment on a cloud hosting service. For this purpose, we have selected an Amazon Web Services (AWS) EC2 t2.micro instance, with the estimated cost calculated at 8.47 USD per month using the AWS Pricing Calculator [38].

Subsequently, a comparative analysis was conducted between Filetherst and other related proposals, based on several criteria, namely community governance features, incentive and disincentive mechanisms, scalability, and data security. A summary of this comparison is presented in Table 2.

**Table 2:** Comparisons of related work with Filetherst.

Criteria	[25]	[26]	[27]	[29]	[31]	[33]	Filetherst
Community governance	No	No	No	No	No	No	Yes
Incentive/Disincentive	Yes^1^	No	No	Yes^1^	No	No	Yes
Scalability	Yes	Yes	Yes	Yes	Yes	Yes	Yes
Data security	Yes	Yes	Yes	Yes	Yes	Yes	Yes

^1^ Only incentive and no disincentive.

Among the proposals we examined as stated in Table 2, the work by Jaiman et al. [29] is the only one that explicitly introduces an incentivisation mechanism. Their approach focuses on encouraging users to share data by rewarding participation; however, the model is limited in scope as it does not incorporate any disincentivisation strategy to address illegal data sharing or discourage malicious activities. In other words, while the system promotes positive behaviour, it lacks a deterrent against undesirable actions.

Another proposal, BMCProtector, takes a slightly different approach by indirectly incentivising content producers. It does so by guaranteeing instant payment for the sale of music or other media content on the network, thereby providing financial motivation for legitimate participation. In addition, it employs a digital watermarking system to trace unauthorised distributions [25]. Although these mechanisms contribute to protecting intellectual property and ensuring fair compensation, the proposal still does not provide a formal disincentivisation model to penalise or prevent harmful actions within the network.

In contrast, the Filetherst platform offers a more comprehensive solution. It implements both incentivisation and disincentivisation through the ETHERST algorithm, thereby not only encouraging desirable behaviours, such as legitimate data sharing, but also actively deterring harmful or illegal activities. This dual approach strengthens the overall governance of the system and ensures a more balanced and secure framework compared to the other proposals. Besides, the backend of Filetherst i.e. ETHERST, proven with the activities simulation of the three type of nodes (good, bad and normal) in a virtual community and analyse and comparison of Average PKIToken balance (APB) and Median PKIToken balance (MPB) make Filetherst a unique solution compare to the other proposals [1].

### Challenges of Filetherst in practical

The idea of Filetherst originated from the common practice among mobile users of sharing files through messaging applications such as WhatsApp or Telegram groups, where users often lack assurance regarding the trustworthiness of the shared content. For example, a group of graphic designers may create a channel to exchange their designs with other designers or clients. Filetherst provides an alternative to conventional WhatsApp or Telegram groups by introducing a secure, self-governance mechanism for sharing data within a community. This approach reduces the likelihood of distributing harmful content, such as viruses or phishing material.

The participation of community members plays a crucial role in evaluating and improving the trustworthiness of shared data. The greater the involvement of legitimate community members, the more accurate the assessment of data reliability becomes. However, one of the challenges we identified is the potential for malicious users to overwhelm the platform with harmful content, thereby undermining the overall performance of Filetherst. While ETHERST mitigates Sybil attacks by employing delayed trust and untrust actions, additional mechanisms are required to prevent the platform from being flooded with malicious data. Without such safeguards, the presence of harmful content could discourage well-intentioned community members from using or joining the platform.

## Conclusions and future work

File-sharing platforms are often flooded with copyright-infringing and harmful files, resulting in income loss for content owners and security risks for users. Filetherst addresses these issues by enabling community members to govern shared content, ensuring trustworthiness through self-regulation supported by ETHERST. This not only mitigates the spread of harmful material but also reduces scam-related activities. Besides, with the support of ETHERST, which promotes the healthy growth of the community, Filetherst provide not only a secure file-sharing platform but also a harmonious virtual community through its unique incentivisation and disincentivisation mechanisms. Finally, we observed that the proof of ownership of shared files is one of the areas in which Filetherst can be improved. For future work, we plan to integrate Ethereum token standards such as ERC-721 (NFTs) to strengthen ownership recognition and further enhance the accuracy of Filetherst in evaluating both users and shared files.
